# Participant and caregiver experiences of an activities of daily living-focused cognitive stimulation program for individuals with mild-to-moderate dementia (CS-ADL)

**DOI:** 10.1177/03080226231225358

**Published:** 2024-01-17

**Authors:** Simone Ryan, Manigandan Chockalingam, Orla Brady

**Affiliations:** 1 Discipline of Occupational Therapy, University of Galway, Galway, Ireland; 2 Primary Care, Health Service Executive (HSE), Ireland

**Keywords:** Dementia, cognitive stimulation, caregiver, qualitative, activities of daily living

## Abstract

**Background:**

Individuals with dementia experience a progressive deterioration in their cognitive and functional abilities, and as a result, require support from informal family caregivers. Non-pharmacological interventions, such as cognitive stimulation, are increasingly being used to address this deterioration. This study explored the participant and caregiver experiences of CS-ADL (Cognitive Stimulation in Activities of Daily Living), an activities of daily living-focused group cognitive stimulation program for individuals with mild-to-moderate dementia.

**Methods:**

A descriptive qualitative design was implemented. CS-ADL participants and caregivers were recruited in dyads through a Psychiatry of Later Life service where CS-ADL was delivered. Semi-structured interviews were completed with each dyad either in-person or via the telephone. Data retrieved were analyzed through reflexive thematic analysis.

**Results:**

CS-ADL was experienced as an acceptable intervention that positively influenced the everyday life of both dyad members, with benefits reported in the memory, mood, daily routine, and social interaction of participants. Furthermore, the facilitation style of group facilitators positively influenced participants’ engagement in CS-ADL. Limited data was gathered regarding the experience of activities of daily living.

**Conclusion:**

This is the first study to explore experiences of CS-ADL, producing a preliminary addition to the evidence-base for CS-ADL. However, further research is required to confirm study findings and explore the experiences of activities of daily living in greater depth.

## Background

Dementia is a clinical syndrome characterized by a progressive deterioration of one or more cognitive domains that interferes with the individual’s social and functional performance (American Psychiatric Association, 2013). The number of people living with dementia worldwide is expected to undergo a threefold increase from 57.4 million people in 2019 to 152.8 million people by 2050 (Nichols et al., 2022). This rapidly growing prevalence places a significant burden on the health and social care systems, thus necessitating the development of effective interventions that address the decline associated with the condition (Arvanitakis et al., 2019).

Cognitive stimulation is a non-pharmacological approach to intervention that aims to enhance global cognitive and social performance through engagement in various activities and discussions (Clare and Woods, 2004). A variety of cognitive stimulation interventions exist across the literature, for example, manualized therapies, group or individualized formats, and cognitive stimulation delivered as part of a wider, multi-component intervention (Ryan and Brady, 2023). Cognitive stimulation interventions have been shown to have significant benefits for cognitive performance, communication, and mood for people living with early-stage dementia; however these benefits do not transfer to the performance of activities of daily living (ADLs; Ryan and Brady, 2023; Woods et al., 2012). ADL performance is one of the main factors affecting quality of life and risk of institutionalization of people living with dementia (Kang et al., 2006). For this reason, a cognitive stimulation intervention addressing ADL performance is essential for mitigating the social and economic impact of dementia, and Cognitive Stimulation in Activities of Daily Living (CS-ADL) is one such intervention.

CS-ADL is a multi-component group cognitive stimulation program for individuals living with mild-to-moderate dementia that aims to enhance ADL performance alongside social and cognitive functioning (Brady and Ryan, 2023). The CS-ADL program was scientifically developed based on the currently available evidence and appropriately revised after it was successfully piloted in an Irish Psychiatry of Later Life service. CS-ADL sessions involve stimulation of a range of ADLs, including basic ADLs such as dressing, grooming, getting out of bed, and instrumental ADLs such as preparing a meal, doing laundry, cleaning and leisure. CS-ADL is facilitated by at least one occupational therapist, with sessions lasting 2 hours, once per week for 7 weeks. A typical group size consists of 5–10 participants. Each session is themed around distinct areas of ADL. Session themes are as follows: “Morning Routine,” “Afternoon Routine,” “Domestic Activities 1,” “Evening Routine,” “Domestic Activities 2,” “Baking,” and “Entertainment and Leisure.” Each session consists of core components, such as cognitive stimulation, physical activity and ADL practice; however the sequence and timing allocated per component may differ between sessions. A typical session begins with introductions, reality orientation, music and discussion. This is followed by a warm-up game that is intended to benefit arousal levels of participants and build rapport among members. The remainder of each session focuses on stimulation and practice of ADLs relevant to the associated theme. For example, the “Morning Routine” session consists of ADL-focused cognitive stimulation activities such as the identification and categorization of morning routine-related objects; discussion and demonstration of assistive devices; discussion and reminiscence of typical morning routines; followed by the planning and preparation of a hot breakfast. ADL practice is completed with materials that attempt to emulate real-life scenarios as much as possible in the confines of the therapy environment. The structure and content of CS-ADL has taken inspiration from previous cognitive stimulation programs (Graessel et al., 2011; Jiménez Palomares et al., 2021; Spector et al., 2011). However, CS-ADL differs from these programs as participants are actively involved in the planning, preparation, and practice of everyday activities. Please refer to supplementary material for more detail. CS-ADL activities are intended to be enjoyable and mentally stimulating for participants, therefore therapists are advised to use their clinical judgment when implementing and adapting activities to match the abilities and needs of group members.

Exploration of participants’ experiences of complex interventions, such as cognitive stimulation, has been recommended by the Medical Research Council to determine the effectiveness of such interventions (Craig et al., 2008). CS-ADL is a newly developed intervention, and no previous research has explored the effectiveness or the experience of taking part in the intervention. Nevertheless, previous research has identified that people living with dementia perceive cognitive stimulation interventions to be a positive experience, with a variety of perceived personal benefits including changes in mood, memory and social connectedness (Spector et al., 2011). However, the cognitive changes associated with dementia mean individuals typically require support to continue engaging in meaningful daily activities, which is generally provided informally by family caregivers (Brodaty and Donkin, 2009; Schulz and Matire, 2004). While caregiving can provide individuals with a sense of fulfillment, it can also lead to various negative personal consequences, such as emotional exhaustion and burnout, which ultimately affects the quality of care provided (Brodaty and Donkin, 2009). However, previous studies have identified that caregivers experience feelings of enrichment and happiness following changes in their care recipients’ mood and communication after attending a cognitive stimulation program (Lauritzen et al., 2022). Evidently, dementia and subsequent treatments affect not only the person diagnosed with it, but they also affect the caregiver. Therefore, exploring the experiences of both people living with dementia who participated in CS-ADL, and their caregivers, will be critical in evaluating the effectiveness of CS-ADL. The research objective for this study is hence to explore the experiences of both participants and their caregivers of CS-ADL.

## Methodology

### Design

This study employed a descriptive qualitative design. This is also an exploratory study, since no previous studies have been conducted on the experience of individuals who have participated in CS-ADL programs.

### Participant selection

A senior occupational therapist who delivered CS-ADL in their practice assisted with recruitment of participants for this study. Participants were recruited in dyads consisting of CS-ADL participants and their caregivers. CS-ADL participants were required to have a formal diagnosis of dementia or major neurocognitive disorder, they must have participated in a CS-ADL program, have sufficient memory of the CS-ADL program, and be able to communicate and hear well enough to participate in an interview. Individuals with significant physical illness, disability or uncontrolled disruptive behaviors that would affect participation in an interview were excluded. Caregiver participants were required to provide informal care for an individual with dementia who has taken part in a CS-ADL program.

Through consideration of the approximate size of a CS-ADL group, previous research of similar cognitive stimulation interventions, and pragmatic factors, a total of 5–10 participant dyads was proposed for recruitment.

### Data collection

Semi-structured interviews were the chosen data collection tool for this study. Three overarching topics composed the interview guide (Table 1). Exploratory questions in relation to the participant and caregivers’ overall experience of CS-ADL and the impact of CS-ADL on their everyday life were chosen. Refer to the supplementary material for the full interview schedule. Interviews were completed with each dyad, consisting of the caregiver and CS-ADL participant. Eligible and consenting participants were provided with the option to be interviewed within their dyad, or separately. Interviews were completed either face-to-face or via telephone and were audio-recorded.

**Table 1.** Interview guide.

Topic	Sample questions
Overall experience of CS-ADL	• “Can you tell me about the type of activities completed during the group?” • “Was there anything you enjoyed/didn’t enjoy about the group?” • “How did you feel meeting people in a similar situation to you?”
Personal impact of CS-ADL	• “Do you feel taking part in CS-ADL affected you personally? If so, in what ways?” • “Did you feel the group benefited your day-to-day life?”
Evaluation of CS-ADL	• “What did you think of the group format?” • “How did you find the length/frequency of sessions?” • “Is there anything you would change about the group?”

### Data analysis

Reflexive thematic analysis (RTA) was the chosen method of data analysis for this study. RTA was completed inductively using six steps as guided by Braun and Clarke (2006). Following data collection, audio recordings were transcribed, anonymized, and uploaded to NVivo 12 Pro software (Lumivero, 2017) for generation of codes and themes. Peer debriefing was completed between the researcher and their academic supervisor to review emerging findings during analysis to enhance credibility of results. The researcher also used reflexive writing exercises throughout each phase of the study to highlight the influence of various dimensions on the research (Supplemental Material).

### Ethical considerations

HSE Research Ethics Committee approval was obtained for this study in 2022. Written informed consent was obtained from all participants to take part in an audio-recorded interview for this study. The Irish Assisted Decision-Making (Capacity) Act (2015) guidelines were followed to facilitate the consent process with cognitively impaired participants.

## Results

Of the six individuals who participated in the CS-ADL program where recruitment took place and their corresponding caregivers, all were deemed eligible and were invited to take part in the study. Four dyads expressed interest in participating. One dyad redacted interest in the study for health reasons, and one CS-ADL participant was excluded due to insufficient memory of CS-ADL. A total of three caregivers and two CS-ADL participants consented to participate and completed an interview with the researcher. One caregiver was interviewed separately from the CS-ADL participant by telephone due to geographical obstacles. All other interviews were conducted in person and in dyads. CS-ADL participant and caregiver data were analyzed through RTA, with three overarching themes identified: “acceptability of CS-ADL,” “effect on everyday life,” and “social connections.” As the sample size of this study is small, and participants may therefore be identified, he/she pronouns have been removed from quotes and replaced with they/them. An ellipsis (. . .) has been used where appropriate to redact repetition of words or to isolate relevant quotations.

### Theme 1: Acceptability of CS-ADL

#### Suitability

All CS-ADL participants and two of three caregivers interviewed reported that activities completed in the group were suitable and meaningful; however, the connotations of suitability differed among participants. One CS-ADL participant stated they enjoyed the elements of music and singing within the group.

I got a very good job; at the very start, I had to sing (CS-ADL Participant 1)

Increased incorporation of music into CS-ADL sessions was suggested.

Maybe ye could put more music into the session (Caregiver 1).

It was also noted that the selection of participants, in relation to certain characteristics such as interests, personality, and stage of dementia, could affect the perceived relevance and outcome of the group. One caregiver expressed the CS-ADL program was more suitable for the care-recipient’s characteristics in comparison to a day-care program they had attended.

. . . I just feel if [CS-ADL] was done more regularly for [CS-ADL Participant 1] anyways it would be much better than a day-care centre, which I don’t think is right for them at the moment. . . (Caregiver 1).

One CS-ADL participant expressed enjoyment of physical games completed in the group and expressed a sense of satisfaction from the opportunity to participate in activities previously completed in their youth.

I like the old activity games. . .It made me feel good like, y’know, when you’re able to kick the ball and knock the skittles down. . . it’s a long time since I kicked a ball (CS-ADL participant 2).

Some caregivers expressed uncertainty regarding the suitability of CS-ADL activities, for reasons such as a lack of communication with the care-recipient, however suggested planning activities in accordance with participants’ interests and activity level.

I don’t know all of what the activities were because [they] never really talked about it, but you know, I’d imagine that not everybody’s as active as [CS-ADL Participant], so . . . I suppose possibly more activity to kind of keep someone like [CS-ADL Participant] engaged (Caregiver 2).

However, not all elements of the group were perceived to be appropriate by all participants, depending on their experience or interest in the activity. CS-ADL participants with little experience in cooking reported they either did not participate or reported feelings of embarrassment when participating in cooking activities.

But I’m a useless cook, so I would just sit and watch (CS-ADL Participant 2).

. . .I was there cooking, and I said to myself, ‘Am I an idiot? (CS-ADL Participant 1).

[They don’t] cook at home (Caregiver 1).

#### Format

The overall structure and timing of CS-ADL sessions was deemed appropriate by participants. However, some participants described the possibility of a greater frequency of sessions being of benefit to group members.

I would just feel if it. . . was done more regularly for [CS-ADL Participant] anyways it would be much better (Caregiver 1).

One CS-ADL participant acknowledged the semi-structured nature of certain elements of the group and reflected on the appreciated opportunities available for participants to change the course of group discussions.

And you weren’t . . . fenced in, y’know that you couldn’t say what you wanted to say and so on . . . the good thing about it was that you could change a situation (CS-ADL Participant 1).

It was suggested that caregivers be involved more in group sessions to ensure adequate carryover from the CS-ADL group intervention sessions to their home environments.

I wonder would it be beneficial to, on one of the sessions, actually bring. . . the person who provides their primary point of contact for care. . .whether it would be useful to have them in on one of the sessions. . . that could be a really nice way of bringing the learning back into the home environment (Caregiver 2).

#### Affective attitude

Overall, participation in CS-ADL was perceived as a positive experience by both members of the dyad. It was noted by caregivers that participants looked forward to each session.

[They’d] always enjoy [the group] because [they] kept down and going and [they were] always ready for me to go out and be at me long before I went to go . . . (Caregiver 3).

CS-ADL participants expressed positive feelings regarding their participation in the group. One participant expressed their appreciation for the activities provided, as they might not have had the opportunity to engage in them outside of the program, due to age-related difficulties.

It’s a good experience. . . to get to talk to people . . . and have the craic with them . . . throw balls and miss the balls. . . it’s not often when you get to our age that you have them activities (CS-ADL Participant 2).

Even though CS-ADL participants reported overall satisfaction with the program, the caregivers’ experience was neither explicitly positive nor negative. Some caregivers believed CS-ADL was a success and were sad to see it end, while other caregivers questioned whether the sessions were beneficial outside of the general enjoyment participants experienced.

I thought [the group] was very successful. . . I was very sorry when it finished (Caregiver 1).

[CS-ADL Participant] never would not enjoy [the group], but what [they] would get out of it would be different (Caregiver 3).

### Theme 2: Impact on everyday life

#### Memory

CS-ADL was perceived by participants as mentally stimulating and challenging for their memory.

[CS-ADL] does challenge your mind alright, because you have to be thinking. . .when someone comes in, oh what’s his name? What’s her name? (CS-ADL Participant 2)

While caregivers acknowledged the mentally stimulating properties of CS-ADL, objective improvements in the CS-ADL participants’ memory were not reported. One caregiver attributed this to the nature of dementia and was doubtful of the potential impact of CS-ADL on a participant’s memory, however, acknowledged that there was no observable degeneration in the care-recipient’s memory.

I’m not sure there were changes in [their] memory to be honest and that’s, I don’t think that’s a reflection on the group, I think it’s a reflection on I suppose where [they’re] at diagnostically. . . I suppose the good part of it was there were no further memory deficits so [their] memory didn’t dis-improve (Caregiver 2).

Nevertheless, caregivers acknowledged how participation in CS-ADL had facilitated their care recipients’ acceptance of their memory deficits.

. . . I think that that’s the positive always of a group that you get that whole spectrum of people who are doing better than you, people that are doing worse than you, people that are roughly the same, and so I think it probably. . . allowed [them] to kind of see that spectrum (Caregiver 2).

#### Mood

Participation in CS-ADL was reported to have a positive impact on the mood of participants. One caregiver described observable changes in the care-recipient’s mood prior to and after attending the group. This in turn had a positive influence on spousal relationships, as the CS-ADL participant reflected on feelings of gratefulness and consideration toward their partner following participation in CS-ADL.

. . . you always came home in very good humour; you know there’d be days where [they] mightn’t go over in very good humour but [they’d] always come back in very good humour (Caregiver 1).

. . . yeah my mood would change. . . on the way home or whatever I’d say ain’t it great I have a lovely [spouse] like [Caregiver 1] looking after me (CS-ADL Participant 1).

#### Communication

Participation in CS-ADL was perceived to provide enhanced opportunities for conversation outside of the group, with CS-ADL participants reported to have an increased drive to engage in conversation with others in their day-to-day life.

Anyone who [they] crossed paths with in the next few days [they] would tell them all about the experience (Caregiver 1).

Within the group, participants also described feeling comfortable to engage with their peers without a fear of judgment.

. . . there was no fear, and that was the great thing about it. . . you could laugh with the girls and. . . the guys and everybody else rather than say oh no I don’t know, I can’t, you know (CS-ADL participant 1).

However, other caregivers did not observe changes in the care-recipient’s communication. One CS-ADL participant speculated that other group members felt feelings of embarrassment which impeded their engagement in group discussion.

[Other group members] wouldn’t talk. You know they really wouldn’t. I say they were embarrassed, embarrassed that they wouldn’t say the right. . . thing or something like that (CS-ADL participant 1).

#### Daily routine

Participation in CS-ADL was described by interviewees as adding purpose to daily life and everyday routines. CS-ADL participants reflected on how a lack of activity in their day-to-day lives and a desire to learn new things prompted their decision to attend the group.

It was the. . .feeling about it that I would be able to learn something from [CS-ADL]. . . and because I was now retired and I was doing very little except doing manual work around here (CS-ADL Participant 1).

. . . what would you be doing only sitting in the house? (CS-ADL participant 2)

Caregivers reiterated this experience, reporting CS-ADL provided an enhanced sense of purpose in daily life for both members of the dyad.

. . . there’s a reason to get up and go out (Caregiver 1).

Despite the short timeframe of each CS-ADL session, one caregiver found that while the care-recipient was attending the group, there were opportunities for the caregiver to take time to complete errands they were otherwise unable to complete in the care-recipient’s presence.

. . . [ the group] wasn’t very long, I could be sort of half ten to half twelve, but yes indeed [they] had little things that you couldn’t really, you didn’t want [them] running around town after you (Caregiver 1).

The COVID-19 pandemic was also described as having a significant impact on the routine of dyads, with the opportunity to engage in CS-ADL reported by caregivers to reduce feelings of isolation for both dyad members following the pandemic.

. . . I’m just trying to bring back the pandemic into our situation because we were thrown together and weren’t able to go anywhere and meet people for such a long time and then it was all a bit depressing (Caregiver 1).

### Theme 3: Social connections

#### Peer relationships

CS-ADL participants reportedly valued and felt encouraged by the social connections formed with their fellow group members, feeling as if their opinions were respected and valued by their peers.

. . . [ the group is] good to be part of . . . you feel that the . . . people that you’re talking to are interested in hearing what you have to say (CS-ADL participant 1).

However, one caregiver reported that their care-recipient did not appear to value the connections made within the group and attributed this to their level of sociability prior to their diagnosis of dementia.

[They] would never talk about anyone else. . . but [they] never [do], [they] had been, all [their] life. . . [they] never [were] a good mixer (Caregiver 3).

Different personalities present in the group were perceived to have an impact on the connections formed between group members. However, some participants appreciated the different perspectives each member brought to the group, noting it enhanced discussion,

. . . some of them didn’t say anything, but some of them. . . they hadn’t a big mouth like me and they kept quiet (CS-ADL Participant 1).

The discussion was very interesting. . . you got different views from everybody (CS-ADL Participant 2).

Caregivers also viewed participation in CS-ADL as an opportunity for the care-recipient to connect with their peers at similar stages of dementia.

. . . to give [them] the opportunity to meet other people who are maybe struggling with the same things that [they were] struggling with (Caregiver 2).

#### Facilitator relationships

One participant reflected on their experience of the relationships formed with group facilitators. Learning about the facilitator’s lives through discussion in the group was deemed as a positive experience.

. . . I was interested what [the facilitators] had to do before they came . . . and they talk to you about their kids, and you know this that and the other (CS-ADL Participant 1)

The CS-ADL participant also felt encouraged by the facilitators to participate in the group, describing how interactions with the facilitators contributed to feelings of belonging within the group, and their level of motivation to engage in certain activities.

They’d ask you a question and so on and so forth and I enjoyed that even though I felt for Christ that I’m getting, y’know, bogged down or whatever, but that’s the type of things was great. . . it made you feel that you were wanted (CS-ADL Participant 1).

#### Link to services

Participation in CS-ADL was also reported to enhance the connections between dyad members and the healthcare services. One caregiver described how the care-recipient’s experience with CS-ADL positively impacted their decision to engage with other group programs available. This in turn provided reassurance to caregivers following a challenging period during the COVID-19 pandemic, when services were significantly reduced.

. . . it was great that [they] got offered [CS-ADL], and now the follow on of getting offered another group, you kind of feel like [they’re] . . . finally linked into services, because for a while, and I know that part of that was COVID, but for a while there was nothing and you were kind of grappling with you know, how [are they] managing? (Caregiver 2)

This caregiver also experienced feelings of reduced burden through knowledge that the care-recipient was being supported by the services.

. . . I just felt like there was somebody checking in on [them]. You know, once a week. . . [they were] doing something of benefit to [them] and also I knew that somebody would phone me if . . . there was any issues (Caregiver 2).

## Discussion and implications

This is the first study to explore the participant and caregiver experiences of CS-ADL, an ADL-focused cognitive stimulation program for individuals with mild-to-moderate dementia. Analysis of data collected from both CS-ADL participants and caregivers yielded three themes in relation to the experience of the intervention: “acceptability of CS-ADL,” “effect on everyday life,” and “social connections.” The findings suggest that CS-ADL is deemed acceptable by both dyad members. Acceptability is a critical factor in the adherence to and success of an intervention and has therefore become a key consideration in the design and evaluation of complex healthcare interventions (Skivington et al., 2021). CS-ADL activities were generally perceived to be suitable and enjoyable for participants, with the timing and format of sessions deemed appropriate by both dyad members. However, some participants suggested an increased frequency of sessions would be beneficial. These findings align with previous literature (Orgeta et al., 2015; Spector et al., 2011), and are a key indicator of the overall effectiveness of the intervention. Nevertheless, some CS-ADL activities, such as cooking, were not deemed appropriate or relevant for certain participants, depending on their experience or interest in the activity. While these participants continued to experience other, general benefits from participating in CS-ADL, recommendations are made to align group activities to the characteristics and preferences of participants. Alignment of group activities to participant preferences may be necessary to maximize the benefit of CS-ADL for ADL outcomes. This could be achieved through individualized goal-setting with patients prior to intervention following the identification of occupational performance problems. Not only would this maximize relevance and benefit for participants, it would maximize therapy time and resources, potentially enhancing implementation of CS-ADL into practice.

However, acceptability is not sufficient on its own in determining the effectiveness of a complex intervention, and the intended outcomes of CS-ADL must also be evaluated. Findings of this study suggest participation in CS-ADL can have a positive effect on the everyday life of both dyad members. CS-ADL was perceived by both dyad members to be mentally stimulating for the CS-ADL participant. However, caregivers did not perceive any improvements in the care-recipient’s memory following participation in CS-ADL, instead reporting that there was no observable deterioration in their memory. This suggests that participation in CS-ADL contributes to the maintenance, rather than improvement, of cognitive functions. This finding aligns with the “use it or lose it” theory that underpins cognitive stimulation; this theory states that enhanced neuronal activity, or “mental stimulation” protects against further cognitive degeneration in dementia, rather than reversing existing deficits (Swaab et al., 2002). Accordingly, cognitive stimulation may not improve cognitive functioning, but rather maintain current cognitive function and delay further decline (Arvanitakis et al., 2019). However, conclusions cannot be drawn regarding the impact of CS-ADL on cognitive functioning without further investigation.

Participation in CS-ADL was also reported to have a positive impact on the mood of participants outside of the group setting. Cognitive stimulation interventions have previously been shown to reduce depressive symptoms and improve the mood of people living with dementia (Chen, 2022). CS-ADL may therefore have potential for reducing depressive symptoms of people living with dementia. Multiple variables may be associated with this outcome. For example, CS-ADL was reported to provide an enhanced sense of purpose to daily life and everyday routines of participants, particularly for dyads emerging from the isolating effects of the COVID-19 pandemic. While a sense of purpose in life has been reported to significantly decrease for older adults following a diagnosis of dementia (Wynn et al., 2020), the pandemic was identified to exacerbate this problem, as it limited the individual’s access to meaningful activities and subsequently contributed to the experience of depressive symptoms (Talbot and Briggs, 2021). While the CS-ADL program was delivered after the lifting of pandemic-related restrictions, participants of this study still reflected on the isolating effect it had on them, and how the opportunity to participate in CS-ADL was a positive addition to their everyday lives post-pandemic. This enhanced sense of purpose therefore could have contributed to the positive changes in mood reported.

Participation in CS-ADL also provided opportunities for people living with dementia to form positive social connections with their peers, with participants deriving value in the companionship and sense of belonging they found through their fellow group members. The opportunities for positive social interactions with peers may have contributed to the positive changes in mood discussed, as increased social engagement had been linked with fewer depressive symptoms in people living with dementia (Beerens et al., 2018). Furthermore, the opportunities for social interactions through CS-ADL may have had a positive impact on the communication of participants, as they were reported to have an enhanced drive to engage in conversation outside of the group, as previously demonstrated through Spector et al.’s (2011) qualitative study.

However, some participants felt different personalities and levels of sociability impacted their social engagement within the group. Group cohesiveness has been identified to contribute to higher rates of intervention engagement (Cole, 2018). While participants are recruited to CS-ADL based on shared diagnostic criteria, factors outside of this commonality must therefore be considered when planning groups. It is recommended that future CS-ADL programs consider both surface-level qualities of participants such as age, ethnicity and gender, alongside deeper-level qualities such as attitudes, values and personalities. Alignment of both qualities has been shown to be predictive of group cohesiveness (Dunlop and Beauchamp, 2011).

Furthermore, the facilitation style of the group facilitators was described as to not only support participants’ engagement but also to enhance their sense of belonging within the group. While the nature of CS-ADL requires facilitators to assume a directive leadership through the pre-selection and structuring of activities, participants described a facilitative approach to leadership that supported their engagement. Facilitative leadership can be described as a collaborative or client-centered approach, which is achieved through the development of a strong therapeutic relationship with group members (Cole, 2018). Facilitators of CS-ADL were perceived to be open with participants, relating to group members through revealing elements of their own personal lives. This approach has been recommended between therapists and people living with dementia, as it enables clients to feel accepted and understood (Yamaguchi et al., 2010). The formation of strong therapeutic relationships appropriate to the client’s needs is therefore considered instrumental in maximizing the therapeutic process (Humbert et al., 2018). While participants in this study valued this dynamic with facilitators, the findings may not be applicable to future CS-ADL participants since successful therapeutic relationships are highly individualized.

While the presence of clinicians to facilitate CS-ADL was valued by participants, the lack of caregiver involvement raised concerns regarding the transferability of learning acquired during CS-ADL sessions to the home environment. Recommendations were therefore made to involve caregivers in at least one session of the program to ensure adequate carryover from CS-ADL sessions to everyday life. Previous literature has demonstrated that caregiver involvement in the delivery of cognitive stimulation enhances caregivers’ awareness of the needs of people living with dementia, thus enabling them to integrate cognitively stimulating activities into the care-recipient’s everyday life (Leung et al., 2017). Therefore, caregiver involvement in CS-ADL should be considered to enhance the transferability of the intervention to everyday life.

While most themes identified were shared experiences between both dyad members, one subtheme, “link to services,” was unique to the caregiver’s experience of CS-ADL. It was reported that the positive nature of CS-ADL encouraged people living with dementia to continue accessing available services. This, in turn, provided reassurance to caregivers that the care-recipient was receiving adequate support, especially following a perceived lack of services during the pandemic. This finding is important, as access to support services not only provides critical opportunities for peer support and enhanced wellbeing for people living with dementia, but also provides reassurance and respite for caregivers (Giebel et al., 2021).

Lastly, CS-ADL aimed to enhance not only the cognitive and social functioning of people living with dementia but also to enhance ADL performance. This is intended to be achieved through participants’ active involvement in the planning, preparation and practice of everyday activities during intervention. While CS-ADL was discussed to influence the everyday lives of participants in relation to their mood, memory and social interaction, minimal data was retrieved with regards to ADL performance outside of the impact on daily routines and the enhanced opportunity to engage in meaningful activities for people living with dementia. While the exploratory nature of this study allowed additional experiences outside of ADLs to be explored, it is likely that minimal data were retrieved on the experience of ADLs due to the small sample size recruited and limitations in instrumentation. Therefore, further, large-scale research is required to explore participant and caregiver perceptions of the effects of CS-ADL on ADL performance in greater depth. Nevertheless, the results of this study provide a valuable preliminary addition to the evidence-base for CS-ADL.

### Limitations

Participant demographics were not gathered for this study as it was deemed inappropriate due to the risk of participant identification considering the small sample size. While the small sample size also limits the credibility of findings, almost half of all participants and their caregivers who took part in the CS-ADL program completed interviews with the researcher. This was deemed adequate due to the exploratory nature of the study; however, further larger scale research is required to enhance confirmability of findings.

While the decision to conduct data collection over differing modalities may have introduced variation in the data, this was not identified during data analysis. In addition, a pre-existing relationship between the researcher and participants may have influenced recruitment and data collection. While this limitation was addressed through researcher reflexivity, the credibility of the study’s findings may still be limited by the potential social desirability bias.

This study is limited by the broad nature of the research question, as limited data regarding the effect of CS-ADL on ADL performance were retrieved. The small sample size further limited these findings. The cognitive impairment of participants may also have limited exploration of the experiences of ADLs, as they may have had limited recall of the specific ADL components of intervention, and instead reflected on the overall feelings ascribed to it. However, as this study was exploratory, the research aimed to identify the overall experience of CS-ADL; therefore further, large-scale research is required to investigate the participant and caregiver experiences of ADLs in greater depth.

## Conclusions

This is the first study to evaluate the participant and caregiver experiences of CS-ADL, an ADL-focused cognitive stimulation program for individuals with mild-to-moderate dementia. The findings of this study indicate that CS-ADL is an acceptable and feasible intervention that positively impacts the lives of both participants and caregivers in a variety of ways. The current study also identified a facilitative approach to group leadership was perceived to support engagement of participants. A variety of suggestions were provided by participants to enhance the acceptability of CS-ADL. However, further large-scale research is required to confirm the outcomes of this study and enhance the transferability of findings.

**Key findings**

- CS-ADL is perceived as an acceptable intervention that positively benefits the daily life of both participants and caregivers.
- A facilitative approach to group leadership positively impacts participants’ engagement.

**What the study has added**

This study has produced a preliminary addition to the evidence-base for an ADL-focused cognitive stimulation program for individuals with mild-to-moderate dementia.

## Supplemental Material

sj-docx-1-bjo-10.1177_03080226231225358 – Supplemental material for Participant and caregiver experiences of an activities of daily living-focused cognitive stimulation program for individuals with mild-to-moderate dementia (CS-ADL) Supplemental material, sj-docx-1-bjo-10.1177_03080226231225358 for Participant and caregiver experiences of an activities of daily living-focused cognitive stimulation program for individuals with mild-to-moderate dementia (CS-ADL) by Simone Ryan, Manigandan Chockalingam and Orla Brady in British Journal of Occupational Therapy

sj-docx-2-bjo-10.1177_03080226231225358 – Supplemental material for Participant and caregiver experiences of an activities of daily living-focused cognitive stimulation program for individuals with mild-to-moderate dementia (CS-ADL) Supplemental material, sj-docx-2-bjo-10.1177_03080226231225358 for Participant and caregiver experiences of an activities of daily living-focused cognitive stimulation program for individuals with mild-to-moderate dementia (CS-ADL) by Simone Ryan, Manigandan Chockalingam and Orla Brady in British Journal of Occupational Therapy

sj-docx-3-bjo-10.1177_03080226231225358 – Supplemental material for Participant and caregiver experiences of an activities of daily living-focused cognitive stimulation program for individuals with mild-to-moderate dementia (CS-ADL) Supplemental material, sj-docx-3-bjo-10.1177_03080226231225358 for Participant and caregiver experiences of an activities of daily living-focused cognitive stimulation program for individuals with mild-to-moderate dementia (CS-ADL) by Simone Ryan, Manigandan Chockalingam and Orla Brady in British Journal of Occupational Therapy
