# Supplementary material for: Participant and caregiver experiences of an activities of daily living-focused cognitive stimulation program for individuals with mild-to-moderate dementia (CS-ADL)
Source: Br J Occup Ther. 2024 Jan 17;87(6):373–82. doi: 10.1177/03080226231225358 (PMC12033870; doi:10.1177/03080226231225358)
Supplement: sj-docx-1-bjo-10.1177_03080226231225358 – Supplemental material for Participant and caregiver experiences of an activities of daily living-focused cognitive stimulation program for individuals with mild-to-moderate dementia (CS-ADL) [file sj-docx-1-bjo-10.1177_03080226231225358.docx]

Table 1- Components of ADLs per session

|  | **Session 1** | **Session 2** | **Session 3** | **Session 4** | **Session 5** | **Session 6** | **Session 7** |
| --- | --- | --- | --- | --- | --- | --- | --- |
| **Theme** | **‘Morning Routine’** | ‘**Afternoon routine’** | **‘Domestic**  **Activities 1’** | ‘**Evening routine’** | ‘**Domestic Activities 2’** | ‘**Baking’** | **‘Entertainment and Leisure’** |
| **Planning** | -Discussion, reminiscence, and sequencing of morning routine and breakfast recipes. | -Discussion and sequencing of getting ready for day. | -Discussion, reminiscence, and sequencing of household chores. | -Discussion and sequencing of evening routine. | -Discussion, reminiscence, and sequencing of grocery shopping, and fruit punch/lemonade recipes. | -Discussion, reminiscence, and sequencing of baking recipes. | -Discussion, reminiscence, sequencing of hobbies and leisure. |
| **Preparation** | -Identification and categorisation of morning routine related items. | -Identification and categorisation of clothes.  -Discussion and preparation of to-do lists. | -Identification and categorisation of household items. | -Preparation of a dinner menu.  -Identification and categorisation of dinner-related items. | -Discussion and preparation of shopping lists/reminders. | -Preparation of a baking recipe.  -Identification and categorisation of baking related items. | -Identification and categorisation of leisure items. |
| **Practice** | -Cooking a fried breakfast. | -Dressing of mannequin.  -Simple gardening activity. | -Hanging clothes on a clothesline.  -Folding and sorting laundry.  -Preparation of a snack. | -Practice of evening routine-related assistive devices.  -Cooking a dinner. | -Grocery shopping calculation activity.  -Preparation of lemonade. | -Baking cookies.  -Cleaning activity. | -Leisure activities. |
