# Supplementary material for: Participant and caregiver experiences of an activities of daily living-focused cognitive stimulation program for individuals with mild-to-moderate dementia (CS-ADL)
Source: Br J Occup Ther. 2024 Jan 17;87(6):373–82. doi: 10.1177/03080226231225358 (PMC12033870; doi:10.1177/03080226231225358)
Supplement: sj-docx-2-bjo-10.1177_03080226231225358 – Supplemental material for Participant and caregiver experiences of an activities of daily living-focused cognitive stimulation program for individuals with mild-to-moderate dementia (CS-ADL) [file sj-docx-2-bjo-10.1177_03080226231225358.docx]

**Interview schedule for:**

**“Participant and caregiver experiences of an ADL-focused cognitive stimulation program for individuals with mild-to-moderate dementia (CS-ADL)”**

**Introduction:**

-Greet participants and thank them for taking part.

-Discuss the CS-ADL program with participants and explain the aims of the research and interview.

**-**Ensure participants have read the information leaflet and signed the consent form.

-Emphasise data from the interview will be treated in a confidential manner and that they can stop the interview at any point without negative consequences.

-Reassure participants that there are no right or wrong answers to the questions, and that if they don’t want to answer a question they are not obliged to.

-Ask participants if they have any questions before beginning.

*Start recording*

**Main themes and follow-up questions**

*Interview questions are in general directed at both caregivers and CS-ADL participants, with minor adjustments required to adapt the questions for each dyad member.*

| **Overall Experience of CS-ADL** | |
| --- | --- |
| **Question** | **Follow-up/Prompt** |
| ‘How did you first find out about the group?’ | ‘*Who approached you about the group?’*  *‘Who told you about the group?’* |
| ‘What made you decide to sign up for the group?’ | *‘What made you think attending the group would be a good idea?’*  *‘What were your expectations?’* |
| ‘Can you tell me about the type of activities completed during the group?’ | *‘Was there music and singing?*  *‘Were there any physical games?’*  *‘Was there cooking/gardening?’*  *‘Can you tell me more about these activities?’*  *‘What was your experience of these activities?’* |
| ‘Was there anything you / [insert name] enjoyed about the group?’ | *‘What did you enjoy about the group?’/ ‘What did [insert name] enjoy about the group?’* |
| ‘Was there anything you / [insert name] didn’t enjoy about the group?’ | *‘What did you not enjoy about the group?’*  *‘Was there anything you found challenging?’/*  *‘Was there anything [insert name] didn’t enjoy about the group?’*  *‘Was there anything [insert name] found challenging?’* |
| ‘How did you feel meeting people in a similar situation to you?’/ How did you feel knowing [insert name] was meeting people in a similar situation to them?’ | *‘How did you feel meeting people who also had challenges with their memory or thinking?’/ ‘How did you feel knowing [insert name] was meeting people who also had challenges with their memory or thinking?’* |

| **Personal Impact of CS-ADL** | |
| --- | --- |
| **Question** | **Follow-up/Prompt** |
| ‘How do you think taking part in the CS-ADL group has affected you personally?’ | *‘Has it helped you?’*  *‘If so, in what ways?’* |
| ‘Did you feel the group benefitted your day-to-day life?’ | *‘If so, in what ways?’* |
| ‘How did you feel knowing that [insert name] was participating in CS-ADL?’ (Directed at caregiver) | *‘Did [insert name]’s participation in the group affect you in any way?’* |
| ‘How do you feel the group affected your / [insert name]’s memory or thinking?’ | *‘Did you notice any changes in your memory or thinking while attending the group?’/ ‘Did you notice any changes in [insert name]’s memory or thinking while attending the group?’*  *‘If so, in what ways?’* |
| ‘Did you notice any changes in how you/ [insert name] engages in daily tasks or routines?’ | *‘If so, in what ways?’*  *‘Were there any changes in how [insert name] participates in activities around the house?’* |
| **Evaluation of CS-ADL** | |
| **Question** | **Follow-up/Prompt** |
| ‘What did you think of the group format?’ | *‘How did you feel being part of a group?’/ How did you knowing [insert name] was a part of a group?’* |
| ‘How did you find the length of each session?’ | *‘Were you happy with the length of the sessions?’*  *‘Were the sessions too long? Too short?’*  *‘Were the sessions tiring?’* |
| ‘What did you think of the overall number of sessions?’ | *‘Were you satisified with how many sessions you were provided?’*  *‘Did you think there were too many/too little sessions? If so, why?’* |
| ‘Was there anything that affected your / [insert name]’s ability to attend the group?’ | *‘Were there any barriers to attending the group?’*  *‘Was there anything that made it difficult to attend?’*  *‘Was there anything that helped you / [insert name] to attend?’* |
| ‘Was there anything you would change about the group?’ | *‘Do you have any recommendations to improve the group?’*  *‘Are there any activities you would change?’*  *‘If so, what would you change?’* |

**Closing:**

**-**Summarise participant’s main thoughts for clarity.

-Ask if there is anything else they would like to contribute or if there is anything else they would’ve liked to discuss.

-Explain how information from the interview will be used.

-Ask participants if would like to be review and approve the interview transcripts/findings, or if they would like to be contacted in future regarding the overall findings of the study.

-Thank participants for participating in the interview.
