# Supplementary figures and images for: Participant and caregiver experiences of an activities of daily living-focused cognitive stimulation program for individuals with mild-to-moderate dementia (CS-ADL)
Source: Br J Occup Ther. 2024 Jan 17;87(6):373–82. doi: 10.1177/03080226231225358 (PMC12033870; doi:10.1177/03080226231225358)

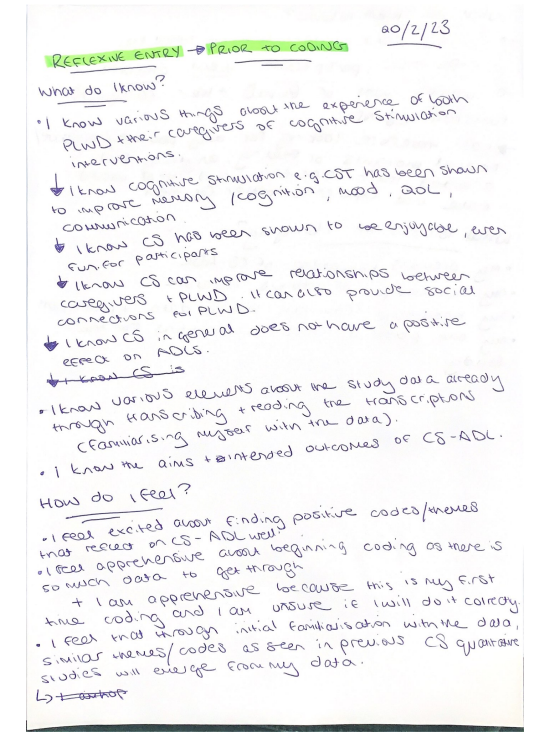


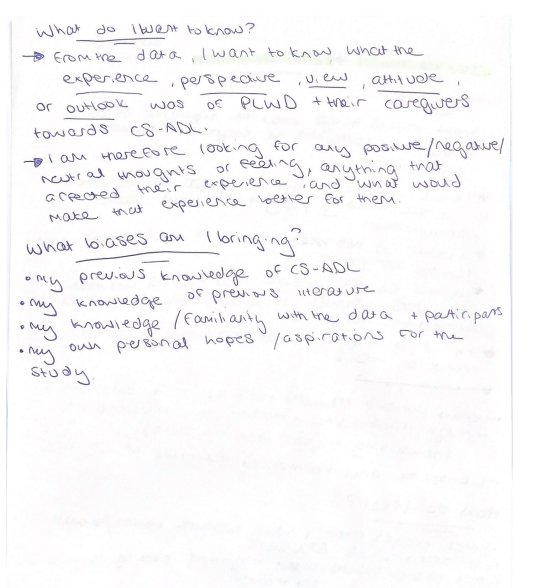

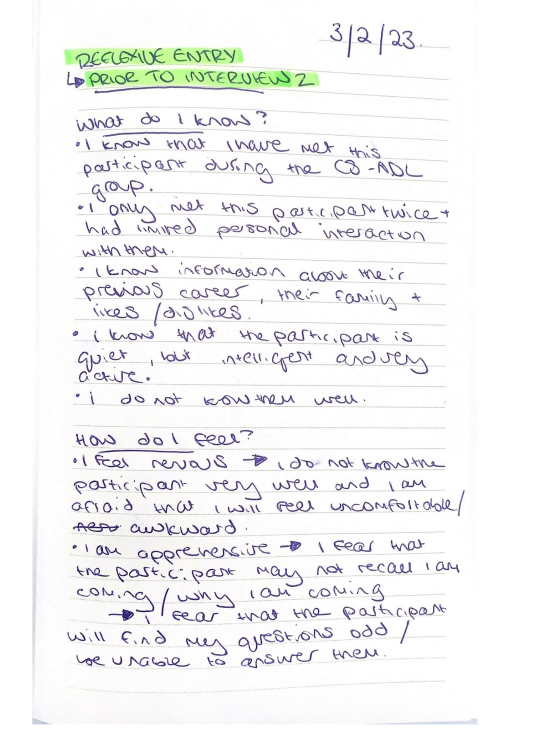


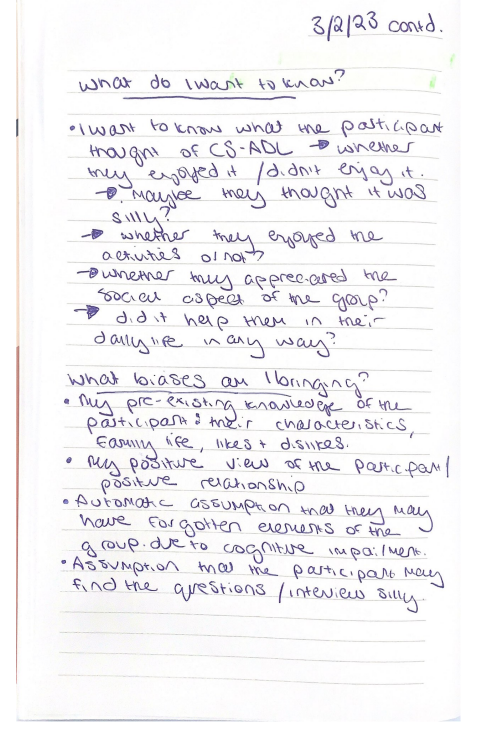

Supplement: sj-docx-3-bjo-10.1177_03080226231225358 – Supplemental material for Participant and caregiver experiences of an activities of daily living-focused cognitive stimulation program for individuals with mild-to-moderate dementia (CS-ADL) [file sj-docx-3-bjo-10.1177_03080226231225358.docx]
